# A cross-sectional survey for the assessment of biosecurity measures in small-scale duck farms in Qalyoubia, Egypt: Comprehensive evaluation and procedural recommendations

**DOI:** 10.14202/vetworld.2023.607-617

**Published:** 2023-03-26

**Authors:** Amany Adel, Hemat S. El-Sayed, Abdelhafez Samir, May F. Abdelaty, Engy A. Hamed, Heba Roshdy

**Affiliations:** 1 Reference Laboratory for Veterinary Quality Control on Poultry Production, Animal Health Research Institute, Agriculture Research Center (ARC), Giza 12618, Egypt; 2 Department of Poultry Diseases, Animal Health Research Institute, Benha-Branch, Agriculture Research Center (ARC), Egypt

**Keywords:** antibiotic resistance, bacterial agents, biosecurity, co-infections, small-scale duck farms, viral diseases

## Abstract

**Background and Aim:**

Biosecurity implementation is fundamental to combating diseases and antibiotic resistance. Therefore, this study aimed to examine the correlation between the implementation of biosecurity measures in small-scale duck farms and the incidence of infectious diseases that threaten the duck industry.

**Materials and Methods:**

Twenty small-scale duck farms of different breeds and production stages were collected as representative samples, focused on two districts in the Qalyoubia governorate, which possesses high-density small-scale farms. A 30-point structured questionnaire was designed to assess the level of biosecurity measures implemented in the sampled farms. These farms were examined for bacterial infection by cultivation, typing, and antibiotic sensitivity tests, in addition to molecular techniques for detecting suspected viral diseases.

**Results:**

The results showed that the farms had high or low levels of biosecurity; only 25% possessed high-level biosecurity. Bacteria, including *Salmonella*, *Escherichia coli*, *Staphylococcus*, and *Pasteurella*, were isolated from all sampled farms. High rates of antimicrobial resistance-reaching up to 100% were observed against some drugs. However, viral causative agents, including HPAI-H5N8, duck viral hepatitis, and goose parvovirus, were isolated from only five farms.

**Conclusion:**

The lack of commitment to biosecurity implementation, particularly personal hygiene, was observed in most sampled farms. Increasing the level of biosecurity reduced the incidence of mixed infections.

## Introduction

Ducks are one of the most important domestic birds and attract the interest of breeders in Egypt. Ducks are a good source of animal protein to fill the nutritional gap, thereby achieving food security. The lack of implementation of biosecurity measures in duck farms is an important reason for the transmission of pathogens, leading to loss of productivity and income [1]. Biosecurity - a set of designed preventive measures applied on farms to control the induction of diseases and minimize the spread of disease within or between farms - is the best effective tool for disease control [2, 3]. The proper understanding and awareness of farmers about biosecurity principles support the implementation of biosecurity measures within farms [4, 5]. These include training for gaining experience [5], being aware of danger and risk [6], and trusting the effectiveness of the disease prevention measure [7]. Sanitation is an important component of biosecurity [8]. However, improper practices in some farms can increase the risk of infection, such as equipment sharing, uncontrolled vehicle movement, and poor personnel biosecurity [9, 10].

Incorporating biosecurity practices into farm design, cleaning, and disinfection may aid in the reduction of bacteria, such as *Salmonella, Escherichia coli, Campylobacter*, and *Staphylococcus aureus*, in poultry farms through environmental contamination during breeding cycles [2]. In addition, biosecurity has a crucial role in quelling the spread of viral diseases in ducks, such as duck viral hepatitis (DVH) and HPAI [1, 11]. Although some studies have discussed the relationship between biosecurity measures and pathogenic biological agents [12, 13], there is a lack of information about the biosecurity status of poultry farms, particularly in small-scale duck farms [14]. Moreover, the biosecurity measures in many farms are not standard and do not meet the required level [15]. Biosecurity practices are commonly applied in large-scale commercial flocks [12, 16], whereas small-scale breeding systems are deficient in biosecurity practices [17, 18].

Therefore, this study aimed to assess the application of biosecurity measures in small-scale commercial duck farms and examine their direct impact on the spread of diseases. In addition, we developed a design for a biosecurity assessment sheet to assist breeders in implementing biosecurity measures.

## Materials and Methods

### Ethical approval

The study proposal was approved by the Animal Care Committee of the Animal Health Research Institute (AHRI), Dokki, Giza, Egypt under protocol number AHRI-2429/8/2020.

### Study period and location

**Figure-1:**
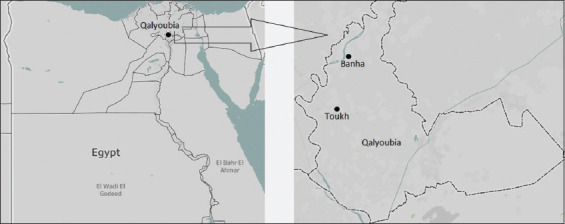
Geographical map of Egypt (Designed in Tableau Public v2020.4 software), illustrates the map of Qalyoubia governorate and the two districts that have been focused in this study, including Toukh and Banha.

The study was based on a mini-survey in small-scale commercial duck farms in and around two districts in Qalyoubia, Egypt from March to September 2021; the geographic illustration was created using Tableau Public v2020.4 software (https://www.tableau.com/support/releases/desktop/2020.4), as shown in Figure-1.

### Sample collection and preparation

Twenty farms that suffered from different clinical signs have been examined as representative samples. All the epidemiological data concerning the farms have been recorded (Supplementary data). The organs of dead and morbid ducks, including liver, spleen, kidney, intestine, tongue, trachea, heart, and lung, have been collected from each farm and transferred on ice to the RLQP, where the organs have been prepared by grinding with sterile sand and phosphate-buffered saline 1% with gentamicin (100 µg/mL). Then, the organs and their homogenates were examined for viral and bacterial suspected agents. The collection and handling of samples have been accomplished under aseptic conditions [19].

### Design of biosecurity implementation questionnaire sheet

A proposed questionnaire was designed to collect data concerning the biosecurity measures collected from farm owners, managers, veterinarians, and workers, as well as the investigator’s observations. The questionnaire sheet included 30 items of biosecurity implementation measures that have been categorized into three main categories (Supplementary data). Farms that recorded scores of more than 50% had high biosecurity, whereas farms that recorded <50% had low biosecurity [20].

### Bacteriological examination

#### Isolation of bacteria

All samples were isolated and biochemically identified for the presence of *Salmonella*, *E*. *coli*, *Staphylococcus*, and *Pasteurella* according to standard methods [21–24].

#### Antimicrobial susceptibility testing

All isolated strains, including *Salmonella*, *E*. *coli*, *Staphylococcus*, and *Pasteurella*, were tested for resistance against a panel of 15 antimicrobial agents (antibiotic discs) using the disk diffusion method (technique), according to the Clinical and Laboratory Standards Institute (CLSI/NCCLS, 2017).

#### Serotyping of E. coli and Salmonella isolates

*Escherichia coli* and *Salmonella* isolates were serotyped by a slide agglutination test using standard *E. coli* antisera (Sifin and Denka Seiken Berlin, Germany) and standard *Salmonella* antisera (Sifin) according to Lee *et al*. [25] and Popoff and Le Minor [26], respectively.

#### Detection of suspected viruses

The suspected diseases examined included avian influenza viruses (H9, H5) in the lung, trachea, and spleen; DVH in the liver; duck enteritis virus (DEV) in the intestine; and Derzi virus in the intestine and tongue.

#### Extraction of the viral genome

Viral genomes were extracted from the prepared homogenates with EasyPure Viral DNA/RNA Kit (Trans, China), following its manual of instructions. The extracted viral genomes were stored at −20°C until use. According to the clinical symptoms and postmortem examination, the targeted viruses were avian influenza viruses (HPAI-H5N8 and LPAI-H9N2), DVH, DEV, and goose parvovirus (Derzy disease).

#### Molecular detection of avian influenza viruses by real-time reverse transcription-quantitative polymerase chain reaction (RT-qPCR)

Samples with avian influenza infection were examined by real-time RT-qPCR. The assay was performed following the instructions of TransScript Probe One-Step qRT-PCR SuperMix (Trans, China). The specific primers for real-time RT-qPCR amplification of H5, N8, and H9 were used (Supplementary data). The reactions were run in Stratagene real-time PCR instrument.

#### Detection of DVH by conventional RT-PCR

The liver from suspected animals was tested using specific primers for DVH, following to the instruction manual of the QuantiTect probe RT-PCR kit (Qiagen, Germany).

#### Detection of DEV and Derzi virus by conventional PCR

The intestines of suspected cases were examined using specific primers for DEV and goose parvovirus (Supplementary data). The PCR reactions were performed using EmeraldAmp Max PCR Master Mix (Takara, Japan), following the manual’s instructions.

### Statistical analysis

The Chi-square test was used for the detection of the significance of variables with Statistical Package for the Social Sciences, version 25 (IBM Corp., NY, USA). The relationships were tested between the implementation of each biosecurity measure and the occurrence of mixed infection and the level of biosecurity in the farms, as well as between the level of biosecurity after evaluation of implementation measure and the occurrence of disease.

Categorical principal components analysis (CATPCA) was applied to the main variables of 30 measures of biosecurity that were applied in each farm under study. The supplementary variables were embedded in CATPCA, including biosecurity levels and three categories of biosecurity measures (infrastructure, hygiene, and control measures). These supplementary variables were used to determine the relationship between the variables [27]. Two-step cluster analysis (TSCA) was obtained from the object scores from CATPCA and was constructed for clustering the farms containing mixed and single infections according to the level of biosecurity.

## Results

### Questionnaire data analysis

According to the biosecurity data in the questionnaire sheet for each farm, the level of biosecurity was classified into two levels. In brief, each item on the questionnaire was assigned one point, and the total number of biosecurity implementations in each farm determined the level of biosecurity. The 20 farms under observation were classified into 12 farms with low biosecurity and eight with high biosecurity. According to the application of the biosecurity measures in our designed sheet, the most applied biosecurity measures in the 20 farms were proper lighting and temperature (95%), visitor restriction (90%), feed quality, and storage (85%). By contrast, the lowest applied measures were wheel disinfectant baths (5%), anteroom or hygiene locks (15%), united overalls and footwear for the workers (20%), availability of disinfectant and detergents (35%), and hand hygiene when handling ducks (35%). The percentage of how far each measure has been applied in farms under the study is presented in Table-1.

**Table-1:** Ratio of implantation of biosecurity points in the twenty duck farms.

Sr. No.	Biosecurity points	Percentage of each point in 20 duck farms
1	Fence around the farm and premises	(8/20) 40
2	Distance between farms (at least 5 kilometers)	(9/20) 45
3	Restricting visitors access	(18/20) 90
4	Separate gate and exit	(9/20) 45
5	Anterooms (hygiene locks)	(3/20) 15
6	Footbath dip at the entry gate	(8/20) 40
7	Wheel disinfectants bath	(1/20) 5
8	United overall and footwear for the workers	(4/20) 20
9	Hand hygiene during handling ducks	(7/20) 35
10	No equipment sharing with other farms	(14/20) 70
11	Isolation of sick birds in a separated area	(13/20) 65
12	Carcasses and used litter disposal (Burial/Burning)	(10/20) 50
13	Cleaning and disinfection of equipment and vehicles	(8/20) 40
14	Absence of wild birds around farm premise	(8/20) 40
15	Absence of rodents, insects and pet animals	(10/20) 50
16	Good litter condition	(12/20) 60
17	Absence of Litter or dead carcass around the farm	(9/20) 45
18	Absence of cracks on walls and roofs of buildings	(13/20) 65
19	The construction of houses are easy to clean and disinfect	(8/20) 40
20	Available disinfectants and detergents	(7/20) 35
21	Absence of rodents and insects inside the farm	(10/20) 50
22	Good food storage	(17/20) 85
23	Good Water source	(11/20) 55
24	Good ventilation	(13/20) 65
25	Proper lighting and temperature degree	(19/20) 95
26	Vaccination programs	(16/20) 80
27	Pet and insecticide control	(8/20) 40
28	Disinfection of drinking water	(0/20) 0
29	Veterinary consultation	(14/20) 70
30	The antibiotic treatment used under veterinarian supervision	(12/20) 60

### Bacterial isolation

*Salmonella*, *E*. *coli*, *Staphylococcus*, and *Pasteurella* strains were isolated and identified from the examined internal organs at 50% (10/20), 55% (11/20), 60% (12/20), and 30% (6/20), respectively.

### Serotyping of *E. coli* and *Salmonella*

Ten strains of *Salmonella* were serologically typed as follows: Three isolates were *Salmonella* Typhimurium; three *Salmonella* Agona, one *Salmonella* Emek, one *Salmonella* Derby, one *Salmonella* Skansen, and one *Salmonella* Warnow (Supplementary data). Eleven strains of *E. coli* were serologically typed as follows: Three O158, two O78, two O91, one O125, one O144, one O44, and one O119 isolate (Supplementary data).

### Antibiotic sensitivity testing

All the isolated bacterial species were tested for antibiotic resistance against 15 antibiotics (Supplementary data). All the isolated bacteria were 100% resistant to penicillin, ampicillin, amoxicillin + clavulanic acid, and sulfamethoxazole. *Staphylococcus* spp. and *Pasteurella* spp. were resistant to 8 out of 15 antimicrobial agents and showed high sensitivity to gentamicin at 100% and 83%, respectively. By contrast, both *E*. *coli* and *Salmonella* possessed variable responses in each sample to the other antibiotics (Supplementary data).

### Molecular detection of suspected viruses

Briefly, farm no. 5 was positive for H5N8 and the virus was detected in the lung, trachea, and kidney by real-time RT-qPCR. The intestines from farms no. 3 and 4 were positive for goose parvovirus (Derzy disease) by conventional PCR. In addition, farm no. 6 and 7 were positive for DVH in congested friable livers. The other farms did not have any viral infection, although they had a bacterial infection (Supplementary data).

### Significance of biosecurity measure application

In this study, we evaluated the level of biosecurity based on the implementation of 30 measures on 20 commercial duck farms (Supplementary data). High-level of biosecurity correlated with a low incidence of infection, especially mixed infection, and *vice versa*. A high-level of biosecurity implementation was recorded in 8 out of 20 farms (Table-2). By contrast, the 12 farms with low levels of biosecurity had mixed infections. A statistically significant relationship was found between a deficiency in biosecurity implementation and the presence of mixed infection in the farms. Increasing the implementation of biosecurity measures limited the spread of viral infections (Table-3). The individual significance of each applied measure is illustrated in Table-4. The significance of the implementation of each biosecurity measure on the level of biosecurity is shown in Table-5. The measures relate to movement restrictions, good ventilation, good water and feed sources, disinfection, and cleaning and have a significant impact on the elevation of biosecurity level.

**Table-2:** Relationship of biosecurity level and occurrence of infections.

Infection	Biosecurity level		Chi-square value	Degree of freedom (df)	Significance

	High	Low			
Mixed infection Single infection	1/8 (12.5%) 7/8 (87.5%)	12/12 (100%) 0/20 (0%)	16	1	0.0001**
Viral infection	0/8 (0%)	5/12 (41.6%)	4.4	1	0.05*
Bacterial infection	8/8 (100%)	12/12 (100%)	Ns	Ns	Ns

Ns=not significant,

* significant values, and

** strong significant values at p-value ≤ 0.05

**Table-3:** An overview of levels and points of biosecurity and the total number of microbes detected in the twenty duck farms.

Biosecurity level	Farm no.	Biosecurity points (%)	Total no. of microbes/farm	Types of microbes
High biosecurity level	1	24 (80)	1	*Salmonella*
	9	22 (73.3)	1	*Staphylococcus*
	10	23 (76.7)	1	*Staphylococcus*
	14	25 (83.3)	1	*Salmonella*
	15	21 (70)	1	*Pasteurella*
	17	24 (80)	1	*E. coli*
	19	23 (76.7)	1	*E. coli*
	18	17 (56.7)	2	*E. coli +* S*taphylococcus*
Low biosecurity level	2	13 (43.3)	2	*Salmonella +* S*taphylococcus*
	3	11 (36.7)	2	*Pasteurella +* Derzi
	6	12 (40)	2	*Staphylococcus +* DVH
	7	14 (46.7)	2	*Salmonella +* DVH
	20	15 (50)	2	*E. coli +* S*taphylococcus*
	4	9 (30)	3	*E. coli* + P*asteurella* + Derzi
	5	5 (16.7)	5	*Salmonella* + E*. coli* + S*taphylococcus* + P*asteurella* + AI (H5N8)
	8	7 (23.3)	4	*Salmonella* + E*. coli +* S*taphylococcus* + P*asteurella*
	11	8 (26.7)	3	*Salmonella* + E*. coli* + S*taphylococcus*
	12	5 (16.7)	4	*Salmonella* + E*. coli* + S*taphylococcus* + P*asteurella*
	13	9 (30)	3	*Salmonella* + E*. coli +* S*taphylococcus*
	16	10 (33.3)	3	*Salmonella* + E*. coli* + S*taphylococcus*

*E. coli*=*Escherichia coli*

**Table-4:** Relationship of application of biosecurity implementations and co-infection.

Biosecurity measures	Chi-square value	Degree of freedom (df)	P-value	Ratio of implementations related to the type of infection			

				Applied		Not applied	
	
				Mixed	Single	Mixed	Single
Fence around the farm and premises	12.85	1	0.0003**	2 (10%)	6 (30%)	12 (60%)	0
Distance between farms (at least 5 kilometers)	10.5	1	0.001**	3 (15%)	6 (30%)	11 (55%)	0
Restricting visitors access	0.95	1	Ns (0.4)	12 (60%)	6 (30%)	2 (10%)	0
Separate gate and exit	10.5	1	0.001**	3 (15%)	6 (30%)	11 (55%)	0
Anterooms (hygiene locks)	8.2	1	0.004**	0	3 (15%)	14 (70%)	3 (15%)
Footbath dip at the entry gate	12.8	1	0.0003**	2 (10%)	6 (30)	12 (60%)	0
Wheel disinfectants bath	0.45	1	Ns (0.5)	1 (5%)	0	13 (65%)	6 (30%)
United overall and footwear for the workers	0.06	1	Ns (0.8)	3 (15%)	1 (5%)	11 (55%)	5 (25%)
Hand hygiene during handling ducks	0.01	1	Ns (0.9)	5 (25%)	2 (10%)	9 (45%)	4 (20%)
No equipment sharing with other farms	3.6	1	0.05*	8 (40%)	6 (30%)	6 (30%)	0
Isolation of sick birds in a separated area	4.6	1	0.03*	7 (35%)	6 (30%)	7 (35%)	0
Carcasses and used litter disposal (Burial/Burning)	3.8	1	0.05*	5 (25%)	5 (25%)	9 (45%)	1 (5%)
Cleaning and disinfection of equipment and vehicles	6.7	1	0.01*	3 (15%)	5 (25%)	11 (55%)	1 (5%)
Absence of wild birds around farm premise	6.7	1	0.01*	3 (15%)	5 (25%)	11 (55%)	1 (5%)
Absence of rodents, insects and pet animals	0.9	1	Ns (0.3)	6 (30%)	4 (20%)	8 (40%)	2 (10%)
Good litter condition	5.7	1	0.01*	6 (30%)	6 (30%)	8 (40%)	0
Absence of Litter or dead carcass around the farm	1.6	1	Ns (0.2)	5 (25%)	4 (20%)	9 (45%)	2 (10%)
Absence of cracks on walls and roofs of buildings	0.84	1	Ns (0.35)	10 (50%)	3 (15%)	4 (20%)	3 (15%)
The construction of houses are easy to clean and disinfect	6.7	1	0.01*	3 (15%)	5 (25%)	11 (55%)	1 (5%)
Available disinfectants and detergents	3.7	1	0.05*	3 (15%)	4 (20%)	11 (55%)	2 (5%)
Absence of rodents and insects inside the farm	3.8	11	0.05*	5 (25%)	5 (25%)	9 (45%)	1 (2%)
Good food storage	1.5	1	Ns (0.2)	11 (55%)	6 (30%)	3 (15%)	0
Good Water source	7	11	0.008**	5 (25%)	6 (30%)	9 (45%)	0
Good ventilation	4.6	1	0.03*	7 (35%)	6 (30%)	7 (35%)	0
Proper lighting and temperature degree	0.4	1	Ns (0.5)	13 (65%)	6 (30%)	1 (5%)	0
Vaccination programs	2.1	1	Ns (0.1)	10 (50%)	6 (30%)	4 (20%)	0
Pet and insecticide control	2.5	1	Ns (0.1)	4 (20%)	4 (20%)	10 (50%)	2 (10%)
Disinfection of drinking water	Ns	Ns	Ns	14 (70%)	6 (30%)	14 (70%)	6 (30%)
Veterinary consultation	3.7	1	0.05*	8 (40%)	6 (30%)	6 (30%)	0
The antibiotic treatment used under veterinarian supervision	5.7	1	0.02*	6 (30%)	6 (30%)	8 (40%)	0

Ns=Not significant,

* significant values, and

** strong significant values at p-value ≤ 0.05

**Table-5:** Relationship of application of biosecurity implementations and the level of biosecurity.

Biosecurity measures	Chi-square value	Degree of freedom (df)	p-value	Ratio of implementation related to the biosecurity level	

				High	Low
Fence around the farm and premises	16.4	1	0.0003**	7/8 (87.5%)	1/12 (8.3%)
Distance between farms (at least 5 kilometers)	15	1	0.0001**	8/8 100%)	1/12 (8.3%)
Restricting visitors access	2.8	1	Ns (0.3)	8/8 (100%)	10/12 (83.3%)
Separate gate and exit	16.3	1	0.0001**	8/8 (100%)	1/12 (8.3%)
Anterooms (hygiene locks)	5.2	1	0.05	3/8 (2.4%)	0/12 (0%)
Footbath dip at the entry gate	12.5	1	0.001**	7/8 (87.5%)	1/12 (8.3%)
Wheel disinfectants bath	1.6	1	Ns (0.4)	1/8 (12.5%)	0/12 (0%)
United overall and footwear for the workers	0.2	1	Ns (1)	2/8 (25%)	2/12 (16.7%)
Hand hygiene during handling ducks	1.3	1	Ns (0.3)	4/8 (50%)	3/12 (25%)
No equipment sharing with other farms	0.2	1	Ns (1)	6/8 (75%)	8/12 (66.7%)
Isolation of sick birds in a separated area	7.2	1	0.02*	8/8 (100%)	5/12 (41.7%)
Carcasses and used litter disposal (Burial/Burning)	3.3	1	Ns (0.2)	6/8 (75%)	4/12 (33.3%)
Cleaning and disinfection of equipment and vehicles	6.8	1	0.02*	6/8 (75%)	2/12 (16.7%)
Absence of wild birds around farm premise	12.5	1	0.002**	7/8 (87.5%)	1/12 (8.3%)
Absence of rodents, insects, and pet animals	3.3	1	Ns (0.2)	6/8 (75%)	4/12 (33.3%)
Good litter condition	4.2	1	Ns (0.07)	7/8 (87.5%)	5/12 (41.7%)
Absence of Litter or dead carcass around the farm	1.6	1	Ns (0.3)	5/8 (62.5%)	4/12 (33.3%)
Absence of cracks on walls and roofs of buildings	1.3	1	Ns (0.35)	4/8 (50%)	9/12 (75%)
The construction of houses are easy to clean and disinfect	12.5	1	0.001**	7/8 (87.5%)	1/12 (8.3%)
Available disinfectants and detergents	6.4	1	0.004*	6/8 (75%)	1/12 (8.3%)
Absence of rodents and insects inside the farm	0.8	1	Ns (0.7)	5/8 (62.5%)	5/12 (41.7%)
Good food storage	2.3	1	Ns (0.2)	8/8 (100%)	9/12 (75%)
Good Water source	11	1	0.001**	8/8 (100%)	3/12 (25%)
Good ventilation	7.2	1	0.02*	8/8 (100%)	5/12 (41.7%)
Proper lighting and temperature degree	0.7	1	Ns (1)	8/8 (100%)	11/12 (91.7%)
Vaccination programs	3.3	1	Ns (0.1)	8/8 (100%)	9/12 (75%)
Pet and insecticide control	0.5	1	Ns (0.6)	4/8 (50%)	4/12 (33.3%)
Disinfection of drinking water	Ns	Ns	Ns	0/8 (0%)	0/12 (0%)
Veterinary consultation	5.6	1	0.04*	8/8 (100%)	6/12 (50%)
The antibiotic treatment used under veterinarian supervision	8.9	1	0.005*	8/8 (100%)	4/12 (33.3%)

Ns=Not significant,

* significant values, and

** strong significant values at p-value ≤ 0.05

**Figure-2:**
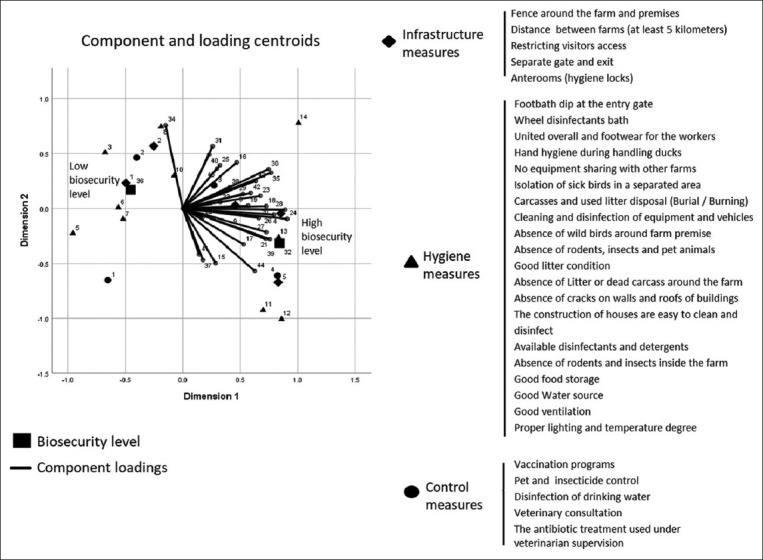
Categorical principal components analysis showed a biplot of component loadings for the active variables (biosecurity measures) and supplementary multiple nominal category variables.

CATPCA illustrates the relationship between the categories of biosecurity measures and the level of biosecurity (Figure-2). The analysis revealed that the intensive application of the control and infrastructure category measures was located in the right quarters, which included high levels of biosecurity variables, indicating a strong relationship between these measures and the level of biosecurity. The left quarters represent a low-level of biosecurity and include hygiene measures that had low scores in our assessment.

**Figure-3:**
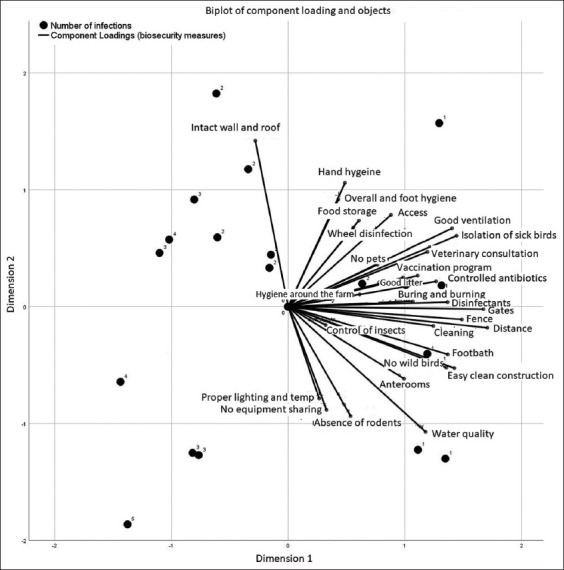
Object scores of the categorical principal components analysis solution and two-step cluster solution. The right quarters include the cluster of high-level security variables with a single infection, while the left quarters contain the cluster of low-level security variables with mixed infections.

A TSCA is shown in Figure-3. The biplot of components (biosecurity measures) and objects (farms with the mixed and single infection variables) illustrated that 8 farms are located in the quarters of a high-level of biosecurity, whereas 12 farms are scattered in the quarters of a low-level of biosecurity.

## Discussion

The study is based on a mini surveillance in the Qalyoubia governorate, which is a focal point for poultry production in Egypt. Although small-scale duck breeding integrates strongly into duck production, we did not have sufficient official data to cover all the farms on this scale. Therefore, our study focused on this type of breeding due to its economic impact and lack of available data. This study assessed the current situation and the implementation of biosecurity measures in 20 small-scale commercial duck farms of different breeds (Supplementary data), based on a purpose-designed questionnaire that encloses 30 points of biosecurity measures recommended in other studies [1, 28]. We found that there was a shortage in the implementation of some key biosecurity measures. The most prevalent shortage was the lack of a water disinfection system in all the farms in this study. Water sanitation plays an important role in reducing disease transmission through farms [3]. Consistently, a deficiency in water sanitation was reported in farms of other species in Egypt [28] and Sudan [29].

Hand washing has a crucial role in disease transmission [30]. The workers’ movement between farm sectors and handling of the equipment without paying attention to hand sanitation may be an important route for microbial dissemination, especially those transmitted orally, such as enteric bacteria [1, 31]. Personal protective equipment (PPE), such as gloves, overalls, and footwear, is a very important biosecurity measure [5]. However, individuals had not committed to PPE in most sampled farms in this study or in other studies on small-scale poultry flocks [8]. Developing countries do not have an awareness of the use of PPE, sanitizers, and disinfectants in farms [32].

Segregation between access and exit points, feet dipping for visitors and workers, disinfection of the vehicles, and a well-prepared anteroom has a statistically significant effect on the reduction of transmission of micro-organisms in the farms in this study, as well as in other studies that have committed to these measures [33, 34]

Equipment sharing without cleaning was identified as a risk factor for transmitting HPAI in chickens [1, 35]. Cleaning and disinfection in our sampled farms significantly impacted the spreading of mixed infections and elevated the biosecurity level. In general, small-scale farms do not pay attention to the cleaning of shared equipment, compared with poultry flocks, as well as that seen in Georgia, United States, where cleaning equipment is cleaned with pressure washers [36].

Personal hygiene, restriction of movement, and presence of an anteroom are important biosecurity items that have reduced the risk of antimicrobial resistance (AMR) strains of *Salmonella* and *E*. *coli* in turkey and broiler farms [37, 38]. In addition, the control measures used to eradicate insects and rodents and prevent contact with wild birds are effective in reducing AMR strains of *Salmonella*, *E. coli* O157, and *Campylobacter* [39, 40]. Wild birds, including migratory birds, are common carriers of pathogens such as avian influenza virus and *Salmonella* spp. [34, 41]. Accordingly, we found that keeping the farms away from the pathway of wild birds is a significantly effective biosecurity measure [5]. Despite the recommendations and awareness, most countries, such as Egypt, USA, and New Zealand, have not completely committed to the mitigation parameters in small-scale flocks that control contact between wild birds and domestic poultry rearing systems, as well as constructing farms near canals, water lands, and wild bird pathways, open rearing systems, and free-roaming of backyard birds [42, 43]. Similarly, the control of rodents, pets, and insects is a critical biosecurity measure, because they play a crucial role in the transmission of viruses, such as avian influenza, and bacteria, such as *Salmonella* spp., *E*. *coli*, and *Pasteurella* [34, 44].

Carcasses, contaminated litter, and sick animals contain a high load of infectious agents and represent a risk of disease transmission [36, 45]. Approximately 50% of the duck farms in our study dispose of carcasses by burying or burning them, causing a significant spread of mixed infections. Some farms condemn carcasses and litter using unhealthy measures, such as discarding them in water pathways around the farms. This causes the spread of pathogens through the contamination of water sources and the surrounding environment [45, 46].

Concerning the construction of the farms, the distance between the farms, the fence around the farms, a good ventilation system, a good quality source of water, and cleanable building materials are significantly important measures in controlling mixed infections and increasing the level of biosecurity. According to Egyptian law (Law No. 906 for 2008), the recommended distance between poultry farms and residential areas should be at least 1 km². This recommended distance is the shortest one that allows for reducing airborne microbial transmission in combination with the surrounding fence [34, 47]. The intact fence is considered the first line of defense that protects the flocks from microbial transmission, as it limits the movement of rodents, pets, and insects in and out of the farms [34, 48], as well as protects against robbery and entering of dirt [5].

Antimicrobial resistance has become a global threat to humans and animals [39, 49]. We report a high rate of AMR in all the detected bacteria against more than 10 antimicrobial drugs (Supplementary data), although 70% of the examined farms committed to a veterinary consultation. Some studies have suggested that the elevation of biosecurity implementation in farms could reduce the need for antimicrobial drug treatments, consequently reducing the risk of antimicrobial-resistant bacteria [39, 50]. The cleaning and disinfection of the equipment with disinfectant play an effective role in reducing the use of antimicrobial medications [51].

Almost all biosecurity parameters impacted the spreading of the most commonly circulating viruses in ducks, such as avian influenza virus, DVH, and parvovirus [52–54]. Vaccination is important for the control of virus infections [55, 56]. In this study, 80% of the sampled farms were committed to regular vaccinations against viral disease; therefore, the examined farms had a low rate of virus detection.

## Conclusion

The results of this study highlight the link between the lack of biosecurity practices in small-scale duck farms and the spreading of infectious diseases in ducks. This mini-survey study reports a deficiency in the application of personal biosecurity measures, and advocates firm application of biosecurity implementation for reducing mixed infections. Moreover, it provides a biosecurity foundation for small-scale duck production that can be used for subsequent studies and risk assessments related to the introduction and spread of dangerous pathogens.

## Recommendations

This study can help farmers to adapt to and improve biosecurity measures in duck farms, even in the event of restrictions, and an attempt to close the gap between farmer knowledge and practices. Therefore, we have some recommendations that could assist in increasing the biosecurity level of small-scale duck farms:

- Pay attention to the national awareness of farmers, breeders, and workers for biosecurity implementations through radio, television programming, training courses, and official interactions.
- Registrations and licenses granted to establish duck farms should be reviewed with an emphasis on the application of biosecurity requirements within these farms.
- Enact laws and regulations that require new farms not to be licensed without applying biosecurity and setting up farms outside residential areas.
- Policymakers should work together to raise awareness and motivate stakeholders in duck farms to implement biosecurity measures to achieve prosperity and protect health.
- A biosecurity implementation questionnaire is recommended for the regular evaluation of biosecurity levels in farms.
- In farms, regular risk assessments should be performed for the critical points based on the biosecurity questionnaire.

## Data Availability

The supplementary data can be available from the corresponding author at a reasonable request.

## Authors’ Contributions

AA: Designed the study, statistical analysis, and drafted the manuscript. HSE, EAH and MFA: Investigation and sample collection. AS and HR: Methodology. All authors have read, reviewed, and approved the final manuscript.
